# Melioidosis with a subdural collection – a case report

**DOI:** 10.1186/s12879-019-3782-0

**Published:** 2019-02-12

**Authors:** H. L. P. Amarasena, F. H. D. S. Silva, P. M. Y. I. Tilakaratna, S. F. Jayamanne, U. K. Ranawaka

**Affiliations:** 1 Professorial Medical Unit, Colombo North Teaching Hospital, Ragama, Sri Lanka; 2 Department of Medicine, Faculty of Medicine, University of Kelaniya, Kelaniya, Sri Lanka

**Keywords:** Melioidosis, *Burkholderia pseudomallei*, Subdural collection, Sri Lanka

## Abstract

**Background:**

Melioidosis is an infection caused by *Burkholderia pseudomallei*, which is more prevalent in the tropics and leads to significant morbidity and mortality. It characteristically produces widespread caseous lesions and abscesses, and can present with varied clinical manifestations. Melioidosis involving the central nervous system is uncommon.

**Case presentation:**

A 42-year-old Sri Lankan male with type 2 diabetes presented with a febrile illness of 6 days with headache and constitutional symptoms. Clinical examination was unremarkable. Four days later, he developed focal seizures involving the left leg and numbness of the left side. Initial laboratory investigations were suggestive of a bacterial infection. Blood culture was reported as positive for a *Pseudomonas* species, which was resistant to gentamicin. Contrast enhanced CT and MRI scans of the brain showed a subdural collection in the right fronto-temporo-parietal region with possible abscess formation. Melioidosis antibody testing using indirect hemagglutination method was reactive with a titre more than 1/10,240.

He was treated with intravenous meropenem and oral co-trimoxazole for 8 weeks (Intensive phase). The subdural collection was managed conservatively, and seizures were treated with oral antiepileptics. At 7 weeks, follow-up contrast enhanced MRI showed improvement of the subdural collection, and inflammatory markers had normalized. He was discharged after 8 weeks, and treated with oral co-trimoxazole and doxycycline for 6 months (eradication phase). At 6 months follow-up, the patient is asymptomatic.

**Conclusions:**

Cerebral melioidosis is an unusual presentation of melioidosis where the diagnosis can be easily missed. Knowledge of the protean manifestations of melioidosis is of paramount importance in order to detect and treat this potentially fatal infection appropriately, especially in tropical countries where the disease is endemic.

## Background

Melioidosis is an infection caused by *Burkholderia pseudomallei*, a facultative intracellular gram-negative organism, previously known as *Pseudomonas pseudomallei* [1]. It is a potentially fatal infection in humans and animals, characterized by widespread caseous lesions and abscesses. Melioidosis of the central nervous system is uncommon, and the predominant presentation is with localized collections in the brain or spinal cord. We report a case of melioidosis with a subdural collection in a Sri Lankan adult male patient.

## Case presentation

A 42-year-old Sri Lankan man presented to the Colombo North Teaching Hospital, Ragama, Sri Lanka (CNTH) with a febrile illness of 6 days, accompanied by headache and constitutional symptoms. He was a grocer from Minuwangoda, a suburban area in the Western Province situated 44 km from Colombo. He was initially investigated and treated at a regional hospital for 4 days, and was transferred to the CNTH for specialized care. He gave a history of type 2 diabetes mellitus for 5 years without microvascular or macrovascular complications, and was a nonsmoker and a teetotaler. There were no specific symptoms suggesting a source of infection such as cough, abdominal pain, urinary symptoms, etc. He was febrile with a temperature of 39.1^0^ C, and examination of the heart, lungs and abdomen was unremarkable. There was no papilloedema, focal neurological signs, pyramidal signs or neck stiffness. Initial laboratory work up revealed features of a bacterial infection, with neutrophil leukocytosis and elevated inflammatory markers (erythrocyte sedimentation rate – 101 mm/1st hour, C-reactive protein - 220 mg/dl). Initial blood cultures done at the regional hospital had yielded an isolate, which was reported as a *Pseudomonas* species; this was sensitive to ceftazidime, imipenem and meropenem and resistant to gentamicin and ceftriaxone. Other basic laboratory investigations including renal and liver function tests, electrolyte panel and urinalysis were normal. Chest x-ray and ultrasound scan of the abdomen were normal, and the trans-thoracic 2-D echo did not show any vegetations. As the unusual antibiotic sensitivity pattern suggested the possibility of melioidosis, blood was sent for serological testing to a specialized Melioidosis Research Laboratory at the Faculty of Medicine, University of Colombo.

**Fig. 1:**
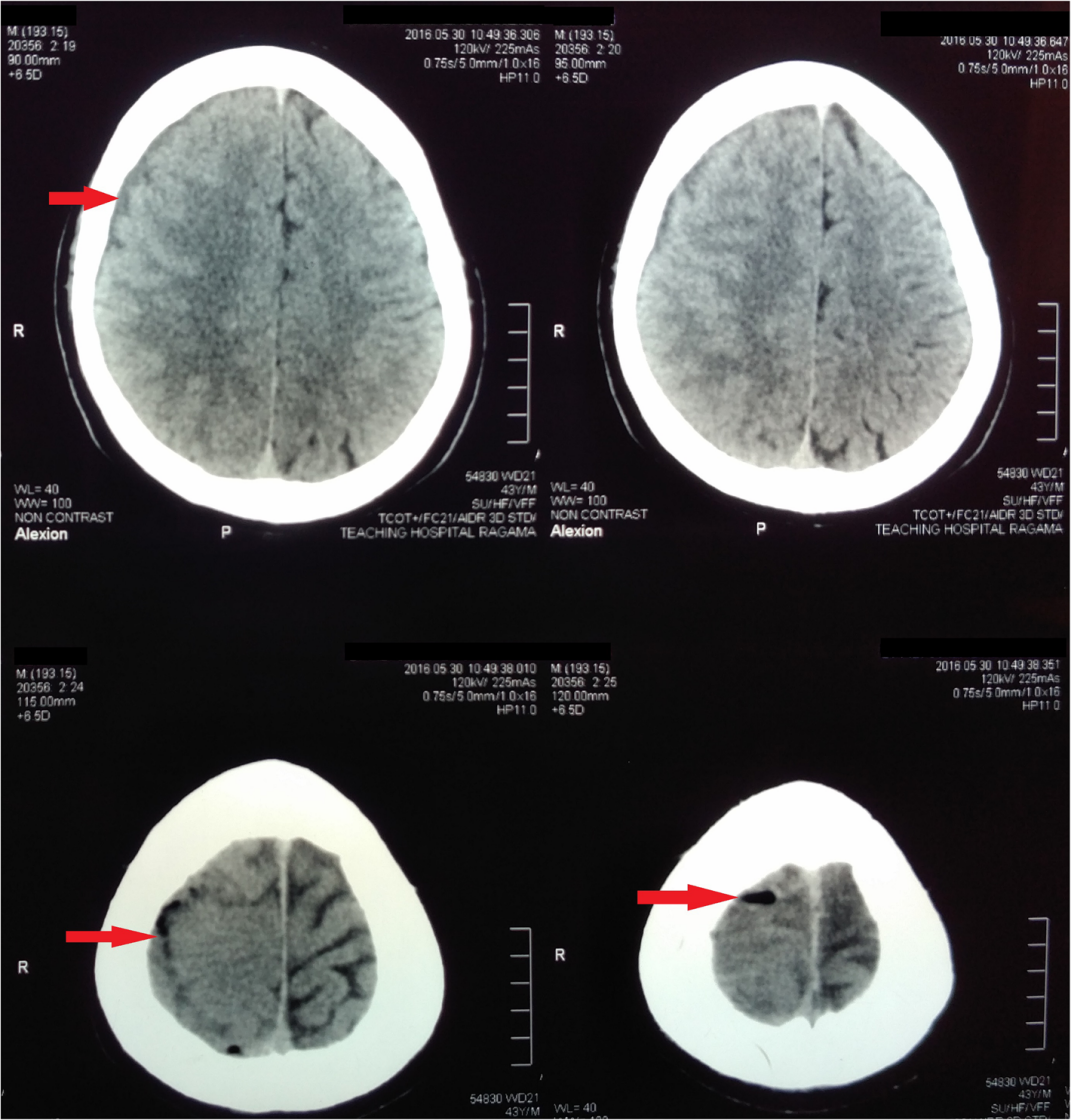
Pre-treatment non-contrast computed tomography of brain showing a right fronto-parietal subdural collection with gas locules

**Fig. 2:**
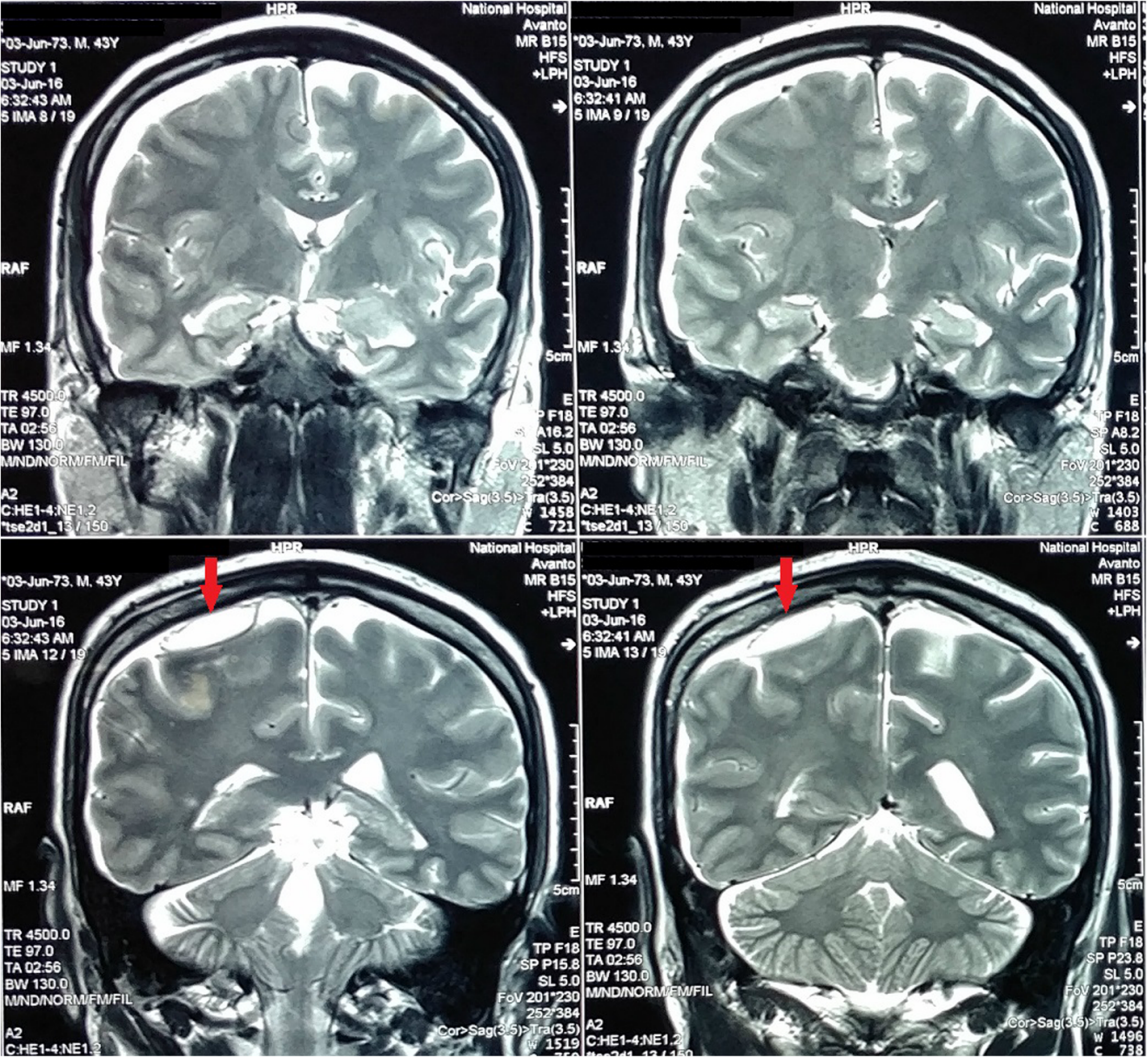
Pre-treatment T2-weighted MRI brain (coronal view) showing a subdural collection in the right fronto-temporo-parietal region with possible abscess formation in the right parietal region

He had been initially treated with intravenous ceftriaxone, and later with ceftazidime according to the antibiotic sensitivity pattern. Although the frequency and intensity of fever spikes reduced with treatment, he continued to have low grade fever and complain of anorexia, malaise and lethargy. On the 4th day after admission to the CNTH (day 10 of the illness), he developed simple partial seizures involving the left lower limb, progressing to persistent numbness of the left side of the body. An urgent CT scan of the head revealed a subdural collection over the right fronto-parietal region with gas locules and obliteration of sulci and gyri, without definite evidence of abscess formation (Fig. 1). Contrast enhanced MRI scan of the brain demonstrated a subdural collection in the right fronto-temporo-parietal region with possible abscess formation in the right parietal region (Fig. 2). Seizures were treated with oral sodium valproate and phenytoin sodium. He was referred for neurosurgical opinion, and the subdural collection was managed conservatively. Results of the indirect hemagglutination assay (IHA) for melioidosis antibodies were received on the following day; an antibody titre of more than 1/10,240 was strongly suggestive of an acute infection with *Burkhoderia pseudomallei* and a diagnosis of cerebral melioidosis was made.

**Fig. 3:**
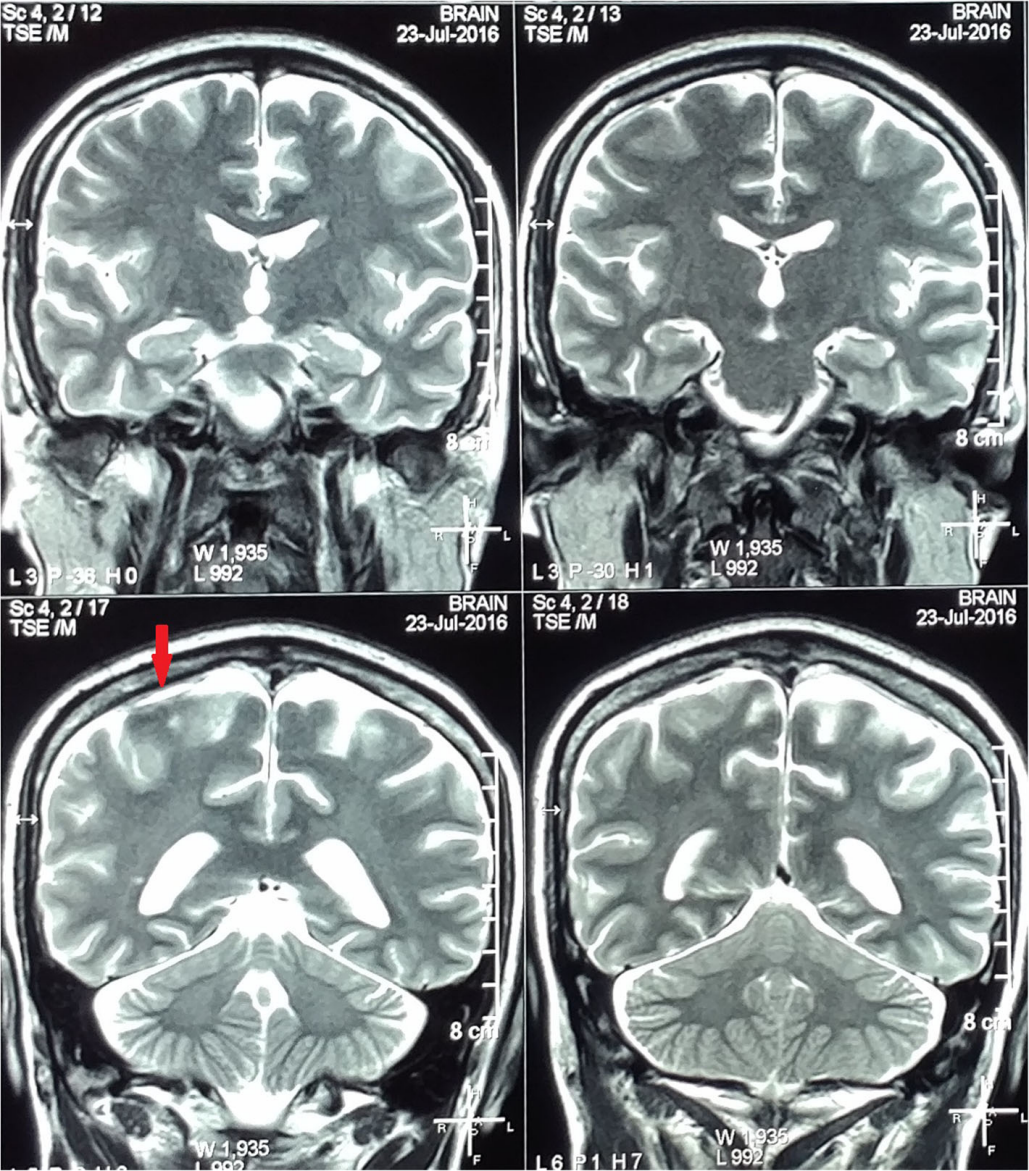
Post-treatment T2-Weighted MRI brain (coronal view) showing resolution of the subdural collection

**Fig. 4:**
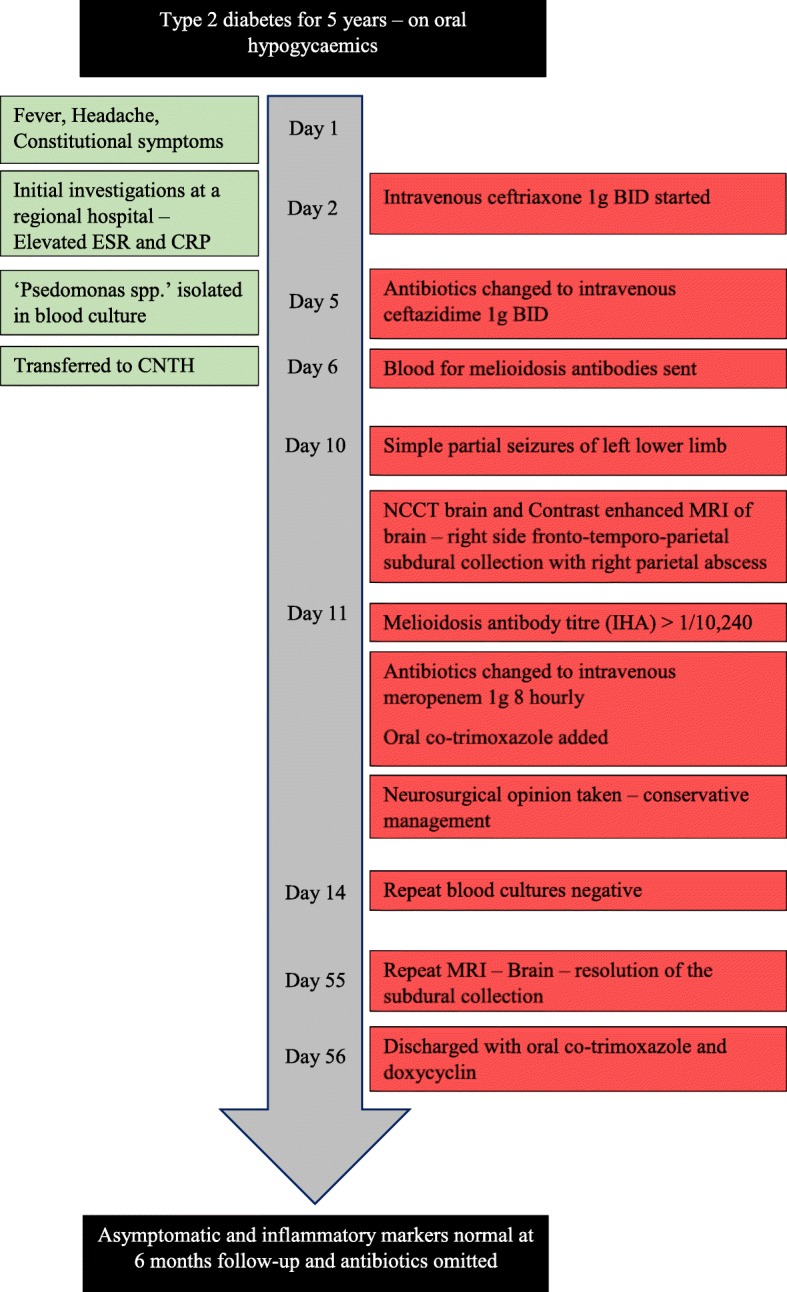
Timeline of events

Antibiotics were changed to intravenous meropenem and oral trimethoprim/sulfamethoxazole (Co-trimoxazole/TMP-SMX). Initial intensive therapy with these antibiotics was continued for 8 weeks, until clinical improvement was evident with resolution of inflammatory markers and radiological improvement confirmed by repeat MRI scan of the brain. (Fig. 3) Repeat blood cultures were sterile after 2 weeks of treatment with antibiotics. There were no further seizures, and the fever and the neurological symptoms resolved completely. He was discharged home with oral TMP-SMX and doxycycline, which were continued for 6 months, and the antiepileptics were gradually tailed off. At 6 months follow up he was asymptomatic. (Fig. 4).

## Discussion and conclusions

We report a patient with melioidosis with a subdural collection, where diagnosis was a challenge mainly due to the delayed onset and unusual nature of the neurological presentation.

The diagnosis was initially suspected based on a blood culture which was reported as ‘positive for *Pseudomonas* spp.’ but with an antibiotic sensitivity pattern which suggested melioidosis. This report highlights the need to actively search for foci of infection when no obvious focus is evident.

Melioidosis is predominantly a disease endemic to tropical regions. It was first reported in 1912 by Whitmore and Krishnaswami, who described 38 cases of a “hitherto undescribed glanders-like illness” in the Rangoon General Hospital, Burma [2]. The highest reported prevalence rates are seen in South Asia, Southeast Asia and Northern Australia [3–6]. South Asia bears 44% of the disease burden, while 40% is shared by the East Asia and Pacific regions [5].

*B. pseudomallei* infection is acquired primarily through percutaneous inoculation [7]. Inhalation is another common mode of acquiring the disease. During severe weather conditions such as monsoon storms, hurricanes, typhoons and cyclones, the organism is released into the air and the predominant mode of transmission shifts from inoculation to inhalation [4]. This was seen following the Indian Ocean tsunami of December 2004, when melioidosis was implicated in the so-called ‘Tsunami Lung’ phenomenon; people swept by tsunami waves developed pneumonia following aspiration of salt water mixed with mud contaminated with bacteria [8–10].

Melioidosis involving the central nervous system (CNS) is rare (seen in about 5%) [11], and can present in many ways including brain and epidural abscesses, encephalomyelitis (predominantly involving the brainstem), aseptic meningitis, dural venous sinus thrombosis and transverse myelitis [4, 12–17]. Isolated subdural collections associated with melioidosis, as seen in our patient, are rarely reported [12–14, 18–20]. A study from Darwin, Australia reported 14 cases with neurological involvement among 540 patients with melioidosis (10 with meningo-encephalitis, 2 with myelitis and 2 with cerebral abscesses); there were no cases of subdural collections [11]. A series of 169 patients with melioidosis in East Malaysia had 3 patients with neurological involvement, and only one of them had a subdural collection which was associated with a brain abscess [12].

The first report of melioidosis from Sri Lanka was in a European tea broker in 1927 [21], and only a few additional cases were reported until recently. However, a laboratory-based case finding program (Sri Lanka National Melioidosis Surveillance Programme) found 32 cases of culture confirmed melioidosis between 2006 and 2014. Cases were reported in 8 of the 9 provinces of the island, with the exception of the Sabaragamuwa Province which consists predominantly of tea and rubber growing highland areas. An increase in incidence was seen with rainfall, especially in the rice growing lowlands, and the annual peak in melioidosis was seen around October when rainfall is highest in the Western Province [22]. The recent increase in reported numbers may well be due to better awareness and detection, but it will be interesting to explore whether the 2004 tsunami that had such a devastating impact on Sri Lanka had any influence on the increase in reported incidence. It is pertinent to note that serological evidence of increased exposure to *B. pseudomallei* was reported from Southern Thailand following the 2004 tsunami, both in tsunami survivors and those unaffected [23].

The first reported case of CNS melioidosis related to Sri Lanka was in a European traveler who developed brain and lung abscesses following a 15-day visit [17]. Cases of cerebral abscess [22], transverse myelitis [16], and Guillain-Barre syndrome [24] have been reported more recently.

Culture is the gold standard in the diagnosis of melioidosis. *B. pseudomallei* grows easily in usual culture media, but can be easily mistaken for *Pseudomonas spp*, especially in non-endemic areas where clinical suspicion of melioidosis would be low and facilities and expertise needed for diagnosis are lacking [4]. The organism is easily outgrown in culture media by faster growing species such as commensals in specimens from non-sterile sites, which can lead to a missed or incorrect diagnosis [25]. The antibiotic sensitivity pattern can provide an important clue to the diagnosis; *B. pseudomallei* is characteristically resistant to the commonly used antimicrobials such as penicillin, ampicillin, first or second-generation cephalosporins, gentamicin, tobramycin and streptomycin [3]. Culturing the organism in Ashdown’s agar containing gentamicin or Ashdown’s broth containing colistin facilitates identification of *B. pseudomallei* [4].

Serology can be helpful in the diagnosis of melioidosis, especially in the presence of high antibody titres. Indirect hemagglutination assay (IHA) is the most widely used serological test, with the sensitivity ranging from 56 to 80% [26, 27] and specificity ranging from 75 to 91% [27, 28]. According to recent data, the background sero-positivity for melioidosis in Sri Lanka is estimated to be 7.4% at a titre of 1/40 [29]. This contrasts with the background sero-positivity in populations with a higher exposure to melioidosis such as Thailand which can be as high as 80% [30] and could have a negative impact on the sensitivity of serological tests.

All patients with melioidosis require an initial intensive therapy with intravenous meropenem, imipenem or ceftazidime for at least 2 weeks. Continuation of intensive therapy for a period of 4 to 8 weeks or longer is recommended for patients who are critically ill or have extensive pulmonary disease, deep seated collections or organ abscesses, osteomyelitis, septic arthritis or neurological melioidosis. Addition of trimethoprim/sulfamethoxazole (TMP-SMX) should be considered for deep seated infections. Drainage of abscesses should also be considered where applicable [4]. It is recommended that the intensive phase is followed by a prolonged period of oral antibiotic therapy of at least 12 weeks to eradicate the organism, but the optimum antibiotic regimen and duration of the eradication phase remain uncertain [31]. Combination therapy with oral TMP-SMX and doxycycline was traditionally recommended [32], but a recent multicenter trial has shown that monotherapy with TMP-SMX for a period of 20 weeks was non-inferior and associated with less adverse events [33].

Melioidosis is not included in the list of notifiable diseases in Sri Lanka, and this should be strongly considered as the incidence of melioidosis appears to be increasing. Melioidosis is a treatable disease, but treatment is difficult and relapses are common [7]. Early recognition is imperative for optimal treatment, but diagnosis can be challenging, as seen in our patient. Facilities for diagnosis should be readily available, especially in the endemic regions. Sri Lanka, and other countries in endemic regions, would do well to invest in providing advanced training for clinicians and microbiologists and developing teams of local experts to ensure delivery of appropriate care. National treatment guidelines should be developed, taking into consideration the availability of facilities and regional variations in disease prevalence. Clearly, more research is needed to understand the differences in epidemiology and transmission patterns across countries. A high degree of suspicion is required to diagnose this evasive illness, and improving awareness on melioidosis among the public and the medical community is of paramount importance.

## Abbreviations

CNS: Central nervous system
CNTH: Colombo North Teaching Hospital
CT: Computed tomography
MRI: Magnetic resonance imaging
TMP-SMX: Trimethoprim/sulfamethoxazole
